# Synopsis of President’s Address of California State Veterinary Medical Association

**Published:** 1895-05

**Authors:** 


### SYNOPSIS OF PRESIDENT'S ADDRESS OF CALIFORNIA STATE VETERINARY MEDICAL ASSOCIATION.

President C. B. Orvis, of the California State Veterinary Medical Association, in his recent address, after referring to the pleasure and gratification at his unanimous election, and paying a just tribute to Secretary Archibald, whose faithful services had done so much for the success and prosperity of their organizarion, spoke of the value of these annual and semi-annual gatherings and of the stimulus they proved to be to all those who made it a duty to attend regularly the meetings of the Association, and of the value specially, of that part of the programme which included practical demonstrations of operations and clinical reports of peculiar cases of importance and interest. He also referred to the strong position among the laity that the profession had gained during the past few years, and specially since the State organization had become so prominent a factor in the work, and of importance in the relation of the profession to the agricultural and stock interests of the State. He also spoke of the generous recognition accorded the veterinary profession by their sister one, the medical branch, and the generous confidence expressed by the State Board of Health in their recognition of work performed by Veterinarians from time to time in conjunction with State work. He congratulated the California Veterinary College on its establishment as well as its adoption of a three years' curriculum. He suggested the wisdom of having an examining board not in connection with the college faculty for the examination of students at the completion of their course for the diploma of the school. He referred to the defeat of the bill establishing State and County Veterinarians with proper quarantine laws, and thought that it was only delayed for a short period of time, and referred to the fact that much information had been spread abroad by veterinarians in regard to certain infectious and contagious diseases, which was unwarranted, and not calculated to enlighten the people as to the dangers associated therewith. He thought that with the valuable diagnostic agent, "mallein," they would soon be able to entirely eradicate glanders from the State if clothed with proper authority.

